# Can gender-fair language modify vocational representations? Assessing the impact of grammatical form and stereotypes on adolescents’ perceived warmth, perceived competence, and anticipated sense of belonging for different occupations

**DOI:** 10.1371/journal.pone.0354871

**Published:** 2026-08-03

**Authors:** Sayaka Sato, Mégane Pittet, Lucie Escasain, Ute Gabriel, Jane Oakhill, Pascal Mark Gygax

**Affiliations:** 1 Department of Psychology, University of Fribourg, Fribourg, Switzerland; 2 Department of Psychology, Norwegian University of Science and Technology, Trondheim, Norway; 3 School of Psychology, University of Sussex, Brighton, United Kingdom; Northumbria University, UNITED KINGDOM OF GREAT BRITAIN AND NORTHERN IRELAND

## Abstract

In recent years, the use of gender-fair language in grammatical gender languages like French has been increasingly encouraged to counteract cognitive biases that arise when the grammatical masculine is used as a generic. Yet little is known about how these language forms affect adolescents for whom vocational interests are still developing. Under this pretext, this study investigates how the use of grammatical masculine and pair forms to describe gender-stereotypical occupations influences adolescents’ vocational representations. Across two preregistered experiments, French-speaking adolescents evaluated perceived warmth, perceived competence, and their anticipated sense of belonging to gender-stereotypical occupations presented in either the masculine or pair forms. Contrary to previous findings among adults, the pair form did not lead to more inclusive perceptions among adolescents. Instead, gender stereotypes associated with the occupation emerged as a primary source for gender representation, especially for girls. Importantly, the effects varied according to adolescents’ gender and age. The implications are discussed in the context of gender-related beliefs and vocational representations.

## Introduction

Adolescence is a sensitive yet critical period characterised by exploring one’s potential career choices [1]. Vocational aspirations and interests during this period are strongly linked to traditional gender role beliefs [2] and gender stereotypes [3,4] as individuals may perceive the competencies or characteristics required for specific occupations as being more aligned with their gendered traits [5,6]. During early adolescence, when young girls and boys embark on gender role exploration, such preconceptions may lead to asymmetries in their career choices.

One aspect shown to influence these gendered vocational choices is the gender cues extant in language. In most grammatical gender languages, such as French, the grammatical masculine form (e.g., *musiciens*_Gr. masculine_ [musicians]) is used as the default form to generically refer to both women and men (*women and men musicians*) while also having a designation specific to men (*men musicians*). Although a standard grammatical rule, using the masculine form as a generic is well documented for driving masculine-biased representations [7–9]. Consequently, this practice hinders the mental accessibility of women in certain professions, leading children to downgrade their perceived likelihood of success [10] and self-efficacy [11,12] for future careers. Gender-fair language (hereon GFL), which grammatically deemphasises the masculine-specific interpretation, has thus increasingly been endorsed as a critical step in countering these effects.

While a growing number of studies on adults have demonstrated the efficiency of GFL in enhancing the mental visibility of women [13–15], research examining adolescents who are progressively internalising gender-related beliefs is still scarce. In the present study, we report two preregistered experiments conducted with adolescents between the ages of 12 and 17, exploring the implications of using GFL, namely the *pair form*, which explicitly uses both the grammatically feminine and masculine forms to emphasise the presence of both genders (e.g., *mécaniciennes*_Gr. feminine_ *et mécaniciens*_Gr. masculine_ [women and men mechanics]). By comparing the pair form with the masculine form, we assess the consequences of using different language forms on adolescents’ social perceptions of gender stereotypical occupations (i.e., warmth and competence) and their anticipated sense of belonging to these occupational groups.

### The influence of the grammatical masculine form and GFL on the mental representation of gender

In most grammatical gender languages, employing the masculine form to refer to both women and men simultaneously is common practice. For example, the masculine form *musiciens* in French denotes either a group composed exclusively of men or a mixed group of women and men musicians. Notwithstanding a potential generic interpretation, extensive psycholinguistic research shows that this use of the masculine form prioritises the mental representation of men, resulting in a masculine bias [16,17]. The masculine form privileges the representation of a specific gender in the mental model because the surface form is strongly linked to the masculine gender, acting as a cognitive cue. Given the probabilistic tendency of the masculine form to refer specifically to men [18], the masculine-specific interpretation is favoured over a generic one.

Prioritising this specific interpretation is not without consequences, as considerable evidence suggests that gender-stereotypical language associating men with masculine words (e.g., competitive) can perpetuate the gender divide in certain occupations [19]. Similarly, using gender-exclusive language, where only one gendered form (e.g., *he*) is used to refer to both genders, has been shown to trigger ostracism among women, resulting in a lower sense of belonging, less motivation, and less expected identification for named professions [20]. Attempts to diminish this masculine bias have consequently led to the adoption of GFL practices that emphasise greater inclusivity. Specifically, GFL essentially employs two main strategies: neutralisation and feminisation [21]. Neutralisation seeks to eliminate gender references by using grammatically gender-neutral linguistic forms. These include epicene nouns, such as *une*_Gr. feminine_/ *un*_Gr. masculine_ *artiste* (an artist), which share the same form, except for the article; collective nouns, such as *le corps*_Gr. masculine_ *étudiant* (the “studying body”, i.e., the student body), which refer to groups of individuals without specifying their gender; and generic nouns like *un individu*_Gr. masculine_ (an individual), which are grammatically gendered but can refer to any person regardless of gender. Feminisation, by contrast, aims to make women linguistically more visible by explicitly mentioning both the grammatically feminine and masculine forms, commonly achieved by using the *pair form* either in its complete (e.g., *les étudiantes et étudi*ants [the women and men students]) or contracted (e.g., *étudiant*·e·s; *étudiant(e)s*) forms. Research has shown that both strategies are effective in attenuating the masculine bias [22–28]. In particular, studies examining the pair form in German [25,29] and French [22,27,30,31] have shown that it can facilitate the mental activation of women, increasing their estimated number in stereotypical occupations [14,22,28,29,32] among adults [14,22,28] and children [11,30].

Despite the reported advantages in cognitively heightening women’s visibility, the use of GFL remains somewhat contested. Beyond the political debates about the readability and practicality of its use [33], the impact of the pair form is not straightforward to interpret. Gender information activated by GFL can interact in intricate ways with gender stereotype information or with preconceptions that a given occupation is for a specific gender (e.g., assuming that *nurse* is a woman’s occupation). Research suggests that enhancing the visibility of women using the pair form has a greater influence on stereotypically masculine occupations (e.g., mechanics) than on stereotypically feminine occupations (e.g., nurses) [14]. For instance, studies in German [29] and French [14] have shown that when participants read texts about a professional gathering, using the pair form instead of the masculine form increased the estimated number of women perceived for masculine-stereotyped occupations, although this was not the case for feminine-stereotyped occupations [14,29]. Arguably, the pair form may heighten the presence of women more for stereotypically masculine than feminine occupations because, by default, feminine-stereotyped occupations are already strongly associated with women.

Insofar as gender stereotypes play a role in the social perception of occupations and interact complexly with language forms, this interplay is particularly relevant for adolescents who are in the process of developing their gender concepts and vocational representations.

### Gender stereotypes and occupational gender representation

According to social role theory, gender stereotypes derive from observations of the distribution of women and men across social roles, including occupations [34,35], and play a central role in decisions related to adolescents’ vocational aspirations [36]. Yet researchers remain divided on the extent to which such stereotypes influence young individuals.

Some argue that girls and boys increasingly internalise gender roles and are confronted by pressure to conform to gender stereotypes during early adolescence [37,38]. In line with this idea, Gottfredson’s circumscription and compromise theory [36] posits that adolescents will evaluate the compatibility of their occupational interests with their self-concept to narrow down options deemed appropriate. As gender is one of the primary aspects of one’s self-concept [36], career choices that are judged inconsistent with one’s gender-role identity are essentially ruled out. As a result, notable differences in occupational perceptions emerge between girls and boys during this period, with each gender group showing less interest in occupations stereotypically associated with the other gender [39,40] and greater confidence in succeeding in occupations stereotypically congruent with their identity [12]. At the same time, others have suggested that adolescents, particularly girls, may develop greater gender flexibility [41], resisting pressures to conform to traditional stereotypes [42]. Despite this growing openness, differences in gendered career interests often persist into late adolescence [39,40]. These mixed findings suggest that it is essential to investigate how adolescents socially perceive members of different occupational groups, an issue that can be understood through two universal dimensions: *warmth* and *competence* [43–45]. The dimension of ‘warmth’ refers to perceived intent, while the dimension of ‘competence’ pertains to the perceived ability. A judgment of high ‘warmth’ entails characteristics considered typically feminine (e.g., friendly), whereas a judgment of high ‘competence’ entails characteristics typically regarded as masculine (e.g., intelligent). Along these lines, people generally assume that feminine characteristics are required for career success in feminine-stereotyped occupations and that masculine characteristics are required for career success in masculine-stereotyped occupations [46].

In recent years, studies have begun to investigate how the use of different GFL forms may modify the social perception of gender-stereotypical occupations along the dimensions of warmth and competence [31,47,48]. Theoretically, when the masculine form is used generically, it may implicitly associate occupations with masculine-associated traits, whereas inclusive GFL forms should promote more equal representations of both genders. Based on this rationale, Vervecken et al. [31] examined how French-speaking adolescents perceive the warmth and competence of individuals in gender-stereotypical occupations when these professions were presented in either the masculine or pair forms. They predicted that the pair form would weaken the impact of stereotypes, leading to success being more equally attributed to women and men, as well as to balanced perceptions of warmth and competence for stereotypically feminine and masculine occupations. In their study, adolescents were orally presented with occupational names and instructed to evaluate each occupation on each measure. The study showed that the pair form indeed led adolescents to perceive success as more equally shared between women and men and to perceive more warmth for masculine-stereotyped occupations. Surprisingly, the same pair form led feminine occupations to be seen as less warm, and language form did not affect perceptions of competence. The authors reasoned that the pair form, which emphasises that occupations can be associated with both genders, led to less differentiated perceptions of warmth and competence for feminine and masculine-stereotyped occupations. Thus, perceptions of warmth increased for masculine occupations and decreased for feminine occupations. As for competence, the authors speculated that because women are no longer seen as incompetent, the linguistic manipulation did not affect perceptions of competence. Horvath et al. [48], although testing adult participants, found somewhat similar results to those of Vervecken et al. [31]. In their study, perceived competence of gender stereotypical occupations among German and Italian adult speakers remained unaffected by the language form used (masculine and pair forms). However, they found that adult Italian-speaking men perceived professions denoted by the pair form as less warm. The authors suggest that this shift in warmth perceptions may be due to the pair form pushing the evaluations towards the middle of the scale.

While Vervecken et al.‘s study [31] highlights the importance of adolescence, when interest in future careers intensifies, and knowledge of grammatical forms deepens, most research on the impact of the pair form has focused on adults. These studies typically examine how the pair form impacts adults’ interpretation of job adverts [32,48] and their mental representations of gender [14,30]. Research on younger populations remains limited, though a handful of studies with children suggest that the pair form can increase the visibility of women [30], boost children’s self-efficacy [11,12], and the perception of success and interests in masculine-stereotyped occupations [10]. Vervecken et al.’s [31] study is among the few (but see also Escasain et al. [49]) to have specifically examined and demonstrated the effect of the pair form among adolescents. This lack of research is noteworthy, as adolescence is a critical developmental stage during which individuals learn and become sensitive to less frequently used GFL forms. Moreover, it is during this period that adolescents actively begin to evaluate social roles [44], making perceptions of warmth and competence in specific occupations central to shaping their future vocational aspirations. In this respect, adolescents may be particularly sensitive to perceptions of whether they might fit in, making their anticipated sense of belonging to future occupations a critical aspect in how vocational interests begin to take form.

### Anticipated sense of belonging

Sense of belonging is considered a fundamental human need [50] and refers to the perception of being accepted or valued as a member of a social group [51]. As individuals seek acceptance from groups that share the social identities that are important to them [52], a sense of belonging, along with social identity, can influence motivation to take on new challenges [53,54] and affect one’s perceived academic potential [54] and interests in pursuing gender-unbalanced academic domains [51]. When one’s sense of belonging is uncertain or questioned, it can foster the belief that they are not welcome in the group, leading to a potential decline in performance and motivation [54]. Concerns about belonging may be particularly problematic for underrepresented group members, such as women, as they may trigger stereotype threat [54]. Importantly, there is some evidence to suggest that language can also influence one’s sense of belonging [20,55]. For example, Stout and Dasgupta found that when confronted with gender exclusive language (*he*) during a mock job interview, women showed a lower sense of belonging than when presented with gender-inclusive (*he or she*) or gender-neutral (*one*) forms [20].

The link between language and sense of belonging is particularly relevant in adolescence, when occupational prospects are often influenced by whether an occupation is dominated by their own gender group [56]. As young individuals grow older and increasingly explore gender roles, their sense of belonging may shift, influencing how they reflect on their future careers. Critically, subtle linguistic cues, such as GFL, may further modify these perceptions, ultimately shaping how they anticipate belonging to specific occupations. Notably, however, a study by Escasain et al. [49] found that presenting health-related occupations in the pair form did not significantly affect adolescents’ sense of belonging, suggesting that linguistic framing alone may not override stereotypes or occupational prestige. Given these mixed findings, examining how GFL affects one’s sense of belonging remains a fundamental area of research.

### Individual factors influencing gender representations

Beyond the impact of specific GFL forms on the gender representation, research has highlighted some individual factors that explain how GFL may or may not exert some influence. For instance, research examining the relationship between attitudes and GFL use [57] indicates that positive attitudes toward GFL are linked to greater openness to inclusive language forms, which may, in turn, foster more inclusive gender representations when GFL is employed [58].

However, research on the relationship between attitudes toward GFL and gender representations is mixed. While Mora et al. [26] found no effects of GFL attitudes on gender representations, Braun et al. [29] showed that participants with positive attitudes toward GFL were more likely to interpret the masculine form as men-specific. According to the authors, because individuals with positive attitudes more frequently use GFL to refer to generic references, the masculine form would lose its signal as a generic reference. In contrast, Tibblin et al. [15] found an opposing finding. In their study, participants with positive attitudes towards GFL perceived fewer women than those with negative attitudes when presented with gender neutral role nouns, independent of whether the masculine or GFL forms were used. The authors argued that those with negative attitudes towards GFL may believe that women are already sufficiently represented in society and thus perceive more women.

The impact of GFL may also depend on adolescents’ stage of career exploration. Career exploration is the first of the three stages in the process leading to a realistic career choice and involves gathering relevant information about oneself and occupations to prepare for an occupation [59]. Research suggests that career exploration contributes to the development of occupational knowledge, which, in turn, affects children’s perceptions of occupational knowledge, particularly in domains they lack knowledge about [60]. For instance, Grotevant et al. [61] showed that the breadth of exploration of potential career choices, particularly the exploration of gender dominance in a given career, predicted adolescents’ career choices that were congruent with their characteristics. These findings suggest that career exploration and the developmental stage adolescents are at may be essential to how adolescents acquire and internalise gender-stereotype knowledge about different occupations. Together, these variables provide an important basis for better understanding the mechanisms by which language forms and gender stereotypes may interact in the context of our study.

### The current study

Although gender stereotypes are generally resistant to change [62,63], research on GFL demonstrates that gendered perceptions of professions can be modified through the explicit use of distinct GFL forms. Such language forms may regulate the prominence of particular gender information, at times making one group more visible than the other, while in other cases, rendering both groups equally accessible. Contrary to adulthood, this aspect is particularly relevant in adolescence, a critical period during which young girls’ and boys’ gender role identities and gender stereotypes evolve, and the influence of these language forms may take different trajectories as individuals grow older. Insofar as language and stereotypes can modify social perceptions of occupations, they may also determine whether adolescent girls and boys differentially imagine themselves in those roles, with potential consequences for their vocational interests and career aspirations. Building on this premise, this study investigates how the use of the masculine and pair forms shapes adolescent girls’ and boys’ social perceptions and sense of belonging to various occupations, and how these effects unfold across distinct developmental stages.

To address these questions, we conducted two preregistered experiments. In the first experiment (Experiment 1), we focused on how the use of the masculine or pair forms to describe gender-stereotyped occupations influences adolescents’ social perceptions of key attributes, namely warmth and competence, as well as their anticipated sense of belonging to these occupations. Conceptually, our goal was to extend Vervecken et al.’s [31] study by revisiting two aspects of their research. First, rather than presenting occupational terms orally, we provided detailed written descriptions of each occupation to ensure adolescents were informed about its functions and responsibilities. Second, we broadened the scope of their study by examining not only the impact of language forms on social perceptions (i.e., perceived warmth and competence) but also on adolescents’ anticipated sense of belonging to the various occupations, a critical factor that should affect how they reflect on their realistic future careers.

To build on these findings, we conducted a second experiment (Experiment 2) to further explore the developmental aspects underlying these effects and to replicate our findings. This follow-up experiment also sought to disentangle the relationship between the various sources of gender information by incorporating individual age-related (i.e., developmental) factors. Specifically, we addressed adolescents’ attitudes toward GFL and their career exploration stage, as realistic vocational choices typically emerge between ages of 11 [64] and 14 [65]. Although Vervecken et al.’s [31] study did not find age-related effects, we took into account the developmental progression of how knowledge of language forms and gender stereotypes evolves as adolescents grow older to be crucial elements of our study, particularly given mixed evidence on their differential impact on girls and boys [39,40]. Accordingly, age and age groupings were factored across both experiments. In addition, because gender stereotypes affect girls and boys differently during this developmental stage, leading to differences in interest [40], perceived success [12], self-efficacy [3], and aspirations [66], we explicitly accounted for adolescents’ gender. On this basis, the present study examined the following hypotheses.

**Hypothesis 1 (H1): Effects of language form and occupational stereotypes on social perceptions.** Our central prediction focused on the effect of language form, which we expected to shape adolescents’ social perceptions by modifying the visibility of women and men in different occupations (H1a). Given that the pair form makes both gender explicit, it should signal greater inclusivity, leading adolescents to view jobs as accessible to both women and men. We expected these effects to operate on the basis that occupations carry gender stereotypes (H1b), and as such, the effect of the pair form should attenuate the gender stereotypicality associated with these occupations. Individuals in feminine-stereotyped and gender-neutral occupations are more typically associated with higher warmth and lower competence, whereas masculine-stereotyped occupations are associated with higher competence and lower warmth. We therefore expected a main effect of occupation stereotype on warmth and competence (H1b), and critically that this stereotype effect would be modulated by language form (language form x occupation stereotype interaction; H1a). Specifically, presenting occupations in the pair form as opposed to the masculine form should lead adolescents to perceive individuals engaged in masculine-stereotyped and gender-neutral occupations as warmer yet less competent, whereas feminine-stereotyped occupations should be viewed as more competent yet less warm.

Note that although some prior studies have found that language form did not affect competence ratings, we predicted that—given our experimental setting—it would still affect our measures of competence. For example, neither Vervecken et al. [31] nor Horvath et al. [48], both of whom examined the influence of the pair form on perceptions of warmth and competence, found evidence that competence ratings were affected [48]. This lack of effect is somewhat surprising given the centrality of competence perceptions in the occupational domain. One potential explanation lies in the differences in the study populations and methodology. Horvath et al. [48], for instance, focused on adult German and Italian speakers, whereas our study targets French-speaking adolescents. As for Vervecken et al. [31], their study presented occupational titles orally to adolescents without providing any job-related descriptions. This lack of information may have limited adolescents’ ability to fully grasp the skills required for occupations, making stereotypical beliefs about the competence required for each job less susceptible to subtle linguistic cues. To address these concerns, the current study provided written, more detailed information about each occupation to assess the potential shift in adolescents’ representations. In doing so, we predicted that the effects of the pair form would also influence competence evaluations, as has been found for warmth perceptions. Additionally, we did not expect gender differences to emerge in our social perception measures but predicted a global effect of language forms (H1a). Although, Vervecken et al. [31] found that girls rated individuals in feminine-stereotyped occupations as warmer than boys, such effects of participant gender have not been consistently demonstrated across studies. We therefore refrained from making directional predictions regarding participant gender.

**Hypothesis 2 (H2): Effects of language form and occupational stereotypes on anticipated sense of belonging.** Regarding anticipated sense of belonging, we predicted language form to influence adolescents’ anticipated sense of belonging to different occupations, although this effect was assumed to vary as a function of adolescents’ gender (H2a). As the pair form more strongly signals the presence of women and men in the occupational role, it should heighten girls’ sense of belonging for different occupations as opposed to the masculine form. In contrast, boys’ belonging [56,67] was not expected to differ by language form as both language forms already highlight the presence of men (H2a). This effect of the pair form was expected to be particularly pronounced for masculine-stereotyped and gender-neutral occupations where women’s visibility is less salient, and weakest for feminine-stereotyped occupations, where women’s presence is already strongly associated with the occupation, and thus less malleable to modification (H2c).

Similar to H1, these hypotheses are grounded on a baseline occupation stereotype effect (H1b), consistent with past findings that individuals are generally more attracted to majors dominated by their gender ingroup [56,67]. Accordingly, we expected both girls and boys to anticipate greater belonging to occupations with stereotypes that are consistent with their gender. Based on this rationale, we expected a main effect of occupation stereotype or a gender by occupation stereotype interaction reflecting a stereotype-congruent anticipated sense of belonging (H2b), modulated by language form depending on participant gender (H2a) and stereotype-occupation stereotype (H2c) as detailed above.

**Hypothesis 3 (H3): Age-related variations in occupational gender stereotypes.** We further expected the influence of occupational stereotypes to vary with adolescents’ age, resulting in an Age x Occupation Stereotype interaction (H3). Prior studies have documented a broad gender difference in vocational interests, with boys and men favouring realistic and investigative domains and girls and women being drawn to artistic and social domains [40,45,57]. These differences are considered to intensify as children grow older and enter adolescence [39,40]. However, while most research characterises adolescence as a phase in which such vocational interests and gender differences stabilise [68], some studies also suggest that these interests are already present in late childhood [69] or that differences in vocational interests may continue to develop in response to environmental factors [57]. For social perceptions of occupations specifically, Vervecken et al. [31] found no age-related variation in gender stereotypes. Given these conflicting findings [40,70], we refrained from making specific predictions about the directionality of these effects.

**Hypothesis 4 (H4): Individual differences in gender representations.** Finally, we expected two individual difference variables that develop during adolescence to disentangle the effects outlined above (H4). First, adolescents’ attitudes toward GFL were expected to explain their susceptibility to language form, such that more open attitudes should be associated with the language form effect. Consequently, we expected a School Year x Participant Gender x Language Form interaction. Second, adolescents’ stages of career exploration were expected to explain the impact of occupational stereotypes. Specifically, more advanced exploration should deepen the knowledge of occupations and their associated stereotypes, resulting in a Career Exploration x Occupation Stereotype interaction.

With these hypotheses, we first tested the core effects of language form and occupational stereotype in Experiment 1.

## Experiment 1

### Method

The study was preregistered on the Open Science Framework (OSF).

#### Participants.

Following Vervecken et al. [31], we set the target sample size at 222 participants; however, the preregistration incorrectly specified 120 participants. As mentioned in the preregistration, we collected more data as the schools provided additional pupils to test. As a result, 346 French-speaking pupils were recruited from two French-speaking schools in Bienne, a French-German bilingual municipality in the Canton of Bern, Switzerland. One school was a lower secondary school consisting of grades 9 (ages 12–13), 10 (ages 13–14), and 11 (ages 14–15), and the second was an upper secondary school consisting of grades 12 (ages 15–16) to 15 (ages 18–20). Participant recruitment and data collection were conducted between June 23 and July 1, 2021.

The research protocol was approved by the Research Ethics Committee at the Department of Psychology of the University of Fribourg (Switzerland). Prior to the data collection day, we communicated detailed information about the study to the participating schools. The information was subsequently shared with the respective teachers, who then informed the pupils in their classes. The teachers did not deem parental consent necessary, as the study was considered part of the curriculum (i.e., career and vocational exploration). Teachers provided written consent to confirm their participation in the study by signing a consent form. The consent form (Experiments 1 & 2) indicated that the research aimed to investigate adolescents’ vocational perceptions and interests.

In line with the participant selection criteria outlined in the preregistration, we first removed 28 participants who did not complete the task properly, as observed by the experimenters, and 37 participants who had never been schooled in French (As Bienne is a bilingual municipality, some parents may choose to school their children in both French and German). We also removed five participants aged 18–20 as they could theoretically be in a transitional phase of adulthood. This decision also follows that of Vervecken et al. (2015) whose participants ranged from 12 to 17 years of age. Finally, as we intended to analyse the effect of participant gender (in terms of girls and boys), we removed 10 participants who identified as “other” as the number was insufficient for a comprehensive analysis. Our final sample comprised 266 participants (162 girls and 104 boys; *M*_age_: 15; *SD*_age_: 1.24 years, range: 12–17 years). Girls and boys did not differ in mean age (*M*_Girls_: 15, *SD*_Girls_: 1.22; *M*_Boys_: 14.9, *SD*_Boys_: 1.25)

#### Stimuli.

All occupations were taken from Misersky et al.’s gender norming study [71], which provided estimates of the gender distribution of around 400 occupations (100% women to 100% men) in different languages. Based on these ratings, we selected 15 occupations in French, comprising five feminine-stereotyped (e.g., *nurses*), five masculine-stereotyped (e.g., *butchers*), and five gender-neutral (e.g., *pharmacists*) occupations. The mean distribution of the selected feminine-stereotyped occupations was 76% (i.e., perceived to be carried out 76% by women and 24% by men). Likewise, the mean distributions for masculine-stereotyped and gender-neutral occupations were 22% and 51%, respectively. Because presenting both grammatically feminine and masculine forms was critical to the experimental manipulation, all selected occupations had grammatically distinct feminine and masculine forms in French. Occupations with identical written forms for both genders, such as *biologistes* [women or men biologists] known as common words [72], were excluded. Additionally, the length of study and training required for each profession was considered to address the perceived prestige of each occupation. This information was taken from the official Swiss website for vocational guidance (orientation.ch, n.d.). We also updated some occupation labels from those listed in Misersky et al. to reflect recent recommendations for accurate labelling (e.g., *mécaniciennes et mécaniciens en maintenance automobile* [women and men automobile maintenance mechanics] instead of *mécaniciennes et mécaniciens auto* [women and men auto mechanics]) [71]. These modifications were based on the Swiss website for vocational guidance (orientation.ch, n.d.).

Each occupation was accompanied by a brief job description taken from the official vocational website of the canton of Vaud in Switzerland (vd.ch/formation, n.d.) detailing the responsibilities of each job (e.g., Nurses: Care and advise people of all ages and look after their well-being. Plan, execute, and coordinate their medical, therapeutic and technical care). None of the descriptions made any explicit reference to gender. To ensure comprehension, we pretested them with 12 participants aged 12–23 who did not participate in the actual experiment. In this pretest, participants underwent the initial experiment and identified any descriptions that might be unclear for 12-year-olds, the youngest population targeted. Based on this pretest, three descriptions (*physician*, *butcher*, *jeweller*) were slightly modified to address the indicated issues.

#### Measures.

Perceived warmth. We used six attributes from Vervecken et al. [31]. The six selected attributes – *amicales* [friendly], *chaleureuses* [warm], *bienveillantes* [benevolent], *dignes de confiances* [trustworthy], *accueillantes* [welcoming], *bien intentionnées* [well intentioned] – were presented in the sentence, *Ce sont des personnes XX.* [They are XX people] (e.g., *Ce sont des personnes amicales.* [They are friendly people]). Responses were rated on a 5-point scale ranging from 1: *Not at all* to 5: *Yes, clearly,* for each attribute. When occupations were presented in the pair form, the attributes were also written in the pair form (e.g., *Ce sont des personnes amicales et amicaux* [They are friendly_Gr. feminine_ and friendly_Gr. masculine_ people]). Cronbach’s alphas for each occupation stereotype were: feminine-stereotyped = .76, masculine-stereotyped = .76, gender-neutral = .76

Perceived competence. Perceived competence for the occupations was assessed using six attributes from Vervecken et al. [31], included in the same sentence and rated on the same 5-point scale as perceived warmth. The selected six attributes included *compétentes* [competent]*, expertes* [expert]*, confiantes* [confident]*, efficaces* [efficient]*, intelligentes* [intelligent]*,* and *performantes* [effective]. The items were intermixed with the warmth items and written in the pair form when the occupations were presented in the pair form. Cronbach’s alphas for each occupation stereotype were: feminine-stereotyped = .79, masculine-stereotyped = .81, and gender-neutral = .78.

Anticipated sense of belonging. We developed five new items to assess participants’ anticipated sense of belonging in a potential future occupation. We based the items on Murphy and Zirkel’s *anticipated educational belonging* and on general adult belonging studies by Baskaya et al. and Cheryan et al. [73–75]. The three questions based on Murphy and Zirkel were “How much would you feel like you belong to the group of people doing this job?”; “If you were doing this job, how comfortable do you think you might feel?”, and “If you were doing this job, to what extent would you feel like you could be yourself?”. The remaining two questions were “Do you think you would get along with other people doing this job?” and “If you were doing this job, to what extent would you be proud of yourself?” We used the word *une personne* [a person] to refer to individuals in these professions because it was more neutral (i.e., epicene) than referring to the occupations in a specific grammatical form or using the word ‘individual’*,* which, in French, has been shown to result in a masculine-biased representation [30]. As with perceived warmth and competence, responses were rated on the same 5-point scale. All questions associated with anticipated sense of belonging were randomised. Cronbach’s alphas were.77 for the feminine-stereotyped,.80 for the masculine-stereotyped and.74 for gender-neutral occupations.

#### Design.

The study took a mixed design with participant gender (girls vs. boys) and language form (masculine vs. pair form) as between-participant variables, and occupation stereotype (feminine vs. masculine vs. gender-neutral) as a within-participant variable. The dependent variables were perceived warmth, perceived competence, and anticipated sense of belonging.

#### Procedure.

The questionnaire was programmed online on the Qualtrics platform (Provo, UT) and accessed by participants via tablets, allowing for group testing. Participants provided general demographic information (e.g., self-identified gender, age, schooling background), followed by the main questionnaire.

The questionnaire presented each occupation individually, accompanied by a job description. Each occupation and description was followed by the ten items measuring perceived warmth and competence, as well as the five items assessing anticipated sense of belonging. Participants responded by marking the appropriate box for each answer. The occupations were presented randomly per participant.

All participants saw only one of the two language forms, with half being presented with the occupations in the masculine form and the other half in the pair form. Participant gender was equally distributed across each language form condition (*n*_Pair–girls_ = 84, *n*_Masculine–girls_ = 78, *n*_Pair–boys_ = 51, *n*_Masculine–boys_ = 53).

#### Data analyses.

We initially preregistered the analyses to fit a linear mixed-effects model with forward stepwise selection to assess adolescents’ perceptions of warmth, competence, and anticipated sense of belonging toward stereotypical occupations. However, we opted for an analytical approach prioritising the maximum model rather than an exploratory forward stepwise selection, as initially planned, because we experimentally manipulated our variables of interest (language form and occupation stereotype) and balanced the design to which these variables were assigned. Nonetheless, for completeness, we also ran the preregistered forward stepwise analyses which can be found on the OSF project page. These analyses yielded the same conclusions as the analyses reported below.

Our main predictors of interest were *participant gender* (girls vs. boys), *language form* (pair form vs. masculine form), and *occupation stereotype* (feminine vs. masculine vs. gender-neutral). Additionally, as linguistic knowledge and understanding of occupational stereotypes develop with age, we also included *participant age* (continuous) as a main predictor to explore its effects. As indicated in the preregistration, we modelled each dependent variable separately, incorporating all four predictors as fixed main effects and all possible interactions. All categorical predictors were sum-to-zero contrast coded (participant gender: girl = −1, boy = 1; language form: masculine form = −1, pair form = 1; occupation stereotype: C1: gender-neutral = −1, feminine = 1, masculine = 0; C2: gender-neutral = −1, feminine = 0, masculine = 1) and participant age was centred at the mean. The random effects structure included by-participant and by-item random intercepts, as well as by-participant random slopes for occupation stereotype and by-item random slopes for language form for all models. The *buildmer()* function from the *buildmer* package [76] was used to identify the maximal feasible model by eliminating non-significant predictors using a backwards-fitting procedure. To determine the significance of fixed effects, *F* and *p*-values were extracted using the ANOVA function of the *lmerTest* package with Satterthwaite’s method [77], and follow-up comparisons were conducted with Tukey corrections using *emmeans* [78]. We also report semi-partial *R*² with 90% confidence intervals for the fixed effects computed with the *r2glmm* package [79]. All data pre-processing and analyses were conducted in the R environment [80], and visualisations were generated with the *ggplot2* package [81] and the *emmip*() function of the *emmeans* package. All model summaries are available on OSF.

### Results

#### Perceived warmth.

**Fig 1:**
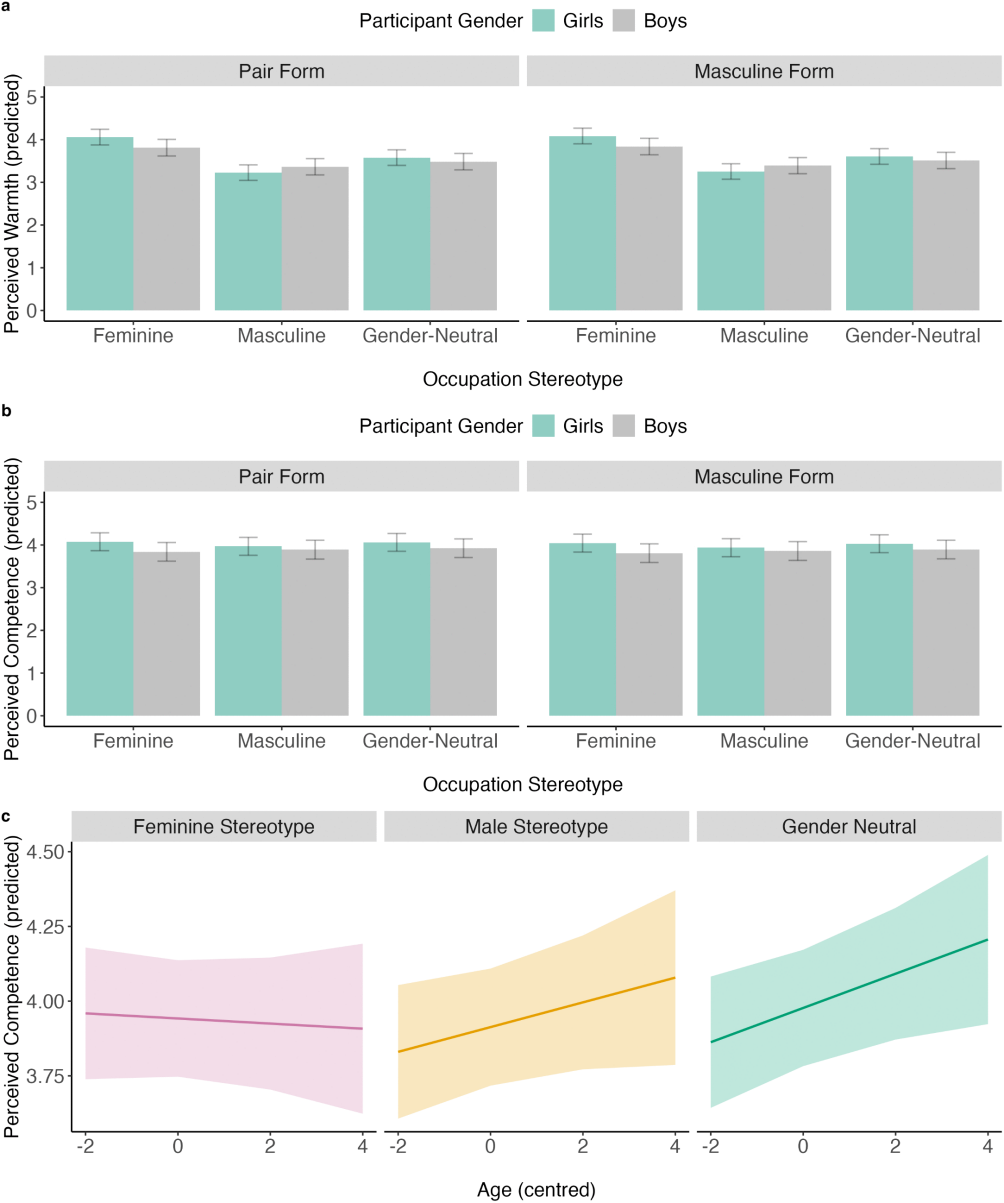
Predicted ratings from Experiment 1: (a) perceived warmth and (b) perceived competence as a function of participant gender, language form, and occupation stereotype, and (c) perceived competence as a function of participant age (centred) and occupation stereotype. Error bars and error bands show confidence intervals.

Beginning with the focal variable, language form was not retained in the final model as it did not improve model fit. Among the retained predictors, occupational stereotype did show a significant main effect, *F*(2, 13.35) = 15.75, *p* < .001, *R*²_β = .078, 90% CI [.071,.084]. Individuals engaged in feminine-stereotyped (*M* = 3.98, *SD* = .92) occupations were perceived as warmer than those in masculine-stereotyped (*M* = 3.29, *SD* = .85; *p* < .0001) and gender-neutral occupations (*M* = 3.56, *SD* = .90; *p* < .001). In contrast, warmth perceptions between masculine-stereotyped and gender neural occupations did not differ (*p =* .1). Additionally, a significant Participant Gender x Occupation Stereotype interaction emerged (see Fig 1a) (*F*(2, 263.82) = 18, *p* < .001, *R*²_β = .007, 90% CI [.005,.010]). To follow-up on this interaction, we compared girls’ and boys’ ratings within each stereotype level, as the measure concerns how perceivers evaluate others. The interaction revealed that while girls perceived individuals engaged in feminine-stereotyped occupations warmer than did boys (Girls: *M* = 4.07, *SD* = 0.83; Boys: *M* = 3.83, *SD* = 1.02; *p* < .001), boys viewed individuals in masculine-stereotyped occupations warmer than did girls (Girls: *M* = 3.24, *SD* = .78; Boys: *M* = 3.38, *SD* = .93; *p* = .029). As for individuals engaged in gender-neutral occupations, girls and boys did not differ in their perceptions of warmth (Girls: *M* = 3.59, *SD* = .85; Boys: *M* = 3.5, *SD* = .97; *p* = .148). No other main or interaction effects were significant.

#### Perceived competence.

Beginning with the key variable, language form did not emerge as a significant main effect, nor did it interact with other variables. The analyses, did, however, reveal a significant main effect of participant gender (*F*(1, 262) = 6.28, *p* = .013, *R*²_β = .006, 90% CI [.004,.008]), which was further qualified by a Participant Gender x Occupation Stereotype interaction, *F*(2, 262.86) = 3.75, *p* = .025, *R*²_β = .001, 90% CI [.00,.002]), (Fig 1b). Similar to perceived warmth, the interaction was decomposed to compare girls’ and boys’ ratings for each stereotype level. The interaction showed that while both gender groups perceived similar levels of competence for individuals taking on masculine-stereotyped occupations (Girls: *M* = 3.95, *SD* = .93; Boys: *M* = 3.87, *SD* = .1; *p* = .26), girls perceived individuals in feminine-stereotyped (Girls: *M* = 4.06, *SD* = .83; Boys: *M* = 3.82, *SD* = 1.03; *p* < .001) and gender-neutral (Girls: *M* = 4.05, *SD* = .88; Boys: *M* = 3.9, *SD* = 1.0; *p* = .038) occupations to be more competent than boys. However, as suggested by the significant Participant Age x Occupation Stereotype interaction (*F*(2, 262.99) = 6.9, *p* < .001, *R*²_β = .001, 90% CI [.001,.003]), participants of different ages had distinct perceptions of competence for individuals in occupations depending on the gender stereotype (Fig 1c). Perceived competence significantly increased for individuals in gender-neutral occupations as adolescents increased in age (*b* = 0.05, *SE* = 0.03, z-ratio = 2.19, *p* = .028), but not for individuals in other occupations. No other main or interaction effects were significant.

#### Anticipated sense of belonging.

**Fig 2:**
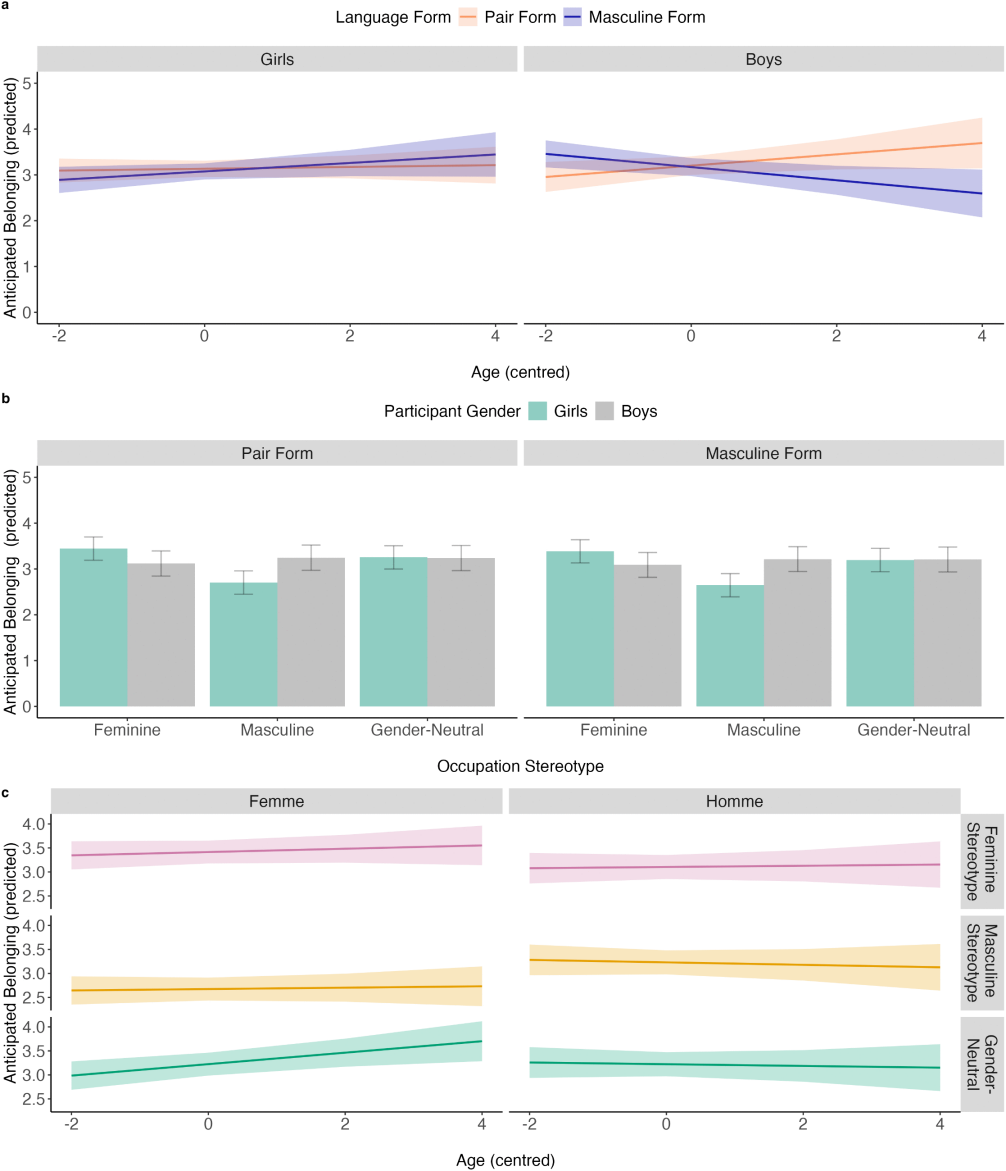
Predicted anticipated sense of belonging ratings from Experiment 1 as a function of (a) participant age (centred), participant gender, and language form, (b) participant gender, language form, and occupation stereotype, and (c) participant age (centred), participant gender, and occupation stereotype. Error bars and error bands represent confidence intervals.

As for sense of belonging, while language form showed no significant main effect, a significant Participant Age × Participant Gender × Language Form interaction emerged, *F*(1, 258) =8.68, *p* = .004, *R*²_β = .007, 90% CI [.005,.01]. The pattern of results revealed that neither the masculine nor the pair forms impacted girls’ anticipated belonging with increasing age (Masculine: *b* = 0.09, *SE* = 0.06, *z*-ratio = 1.6, *p* = .109; Pair: *b* = 0.02, *SE* = 0.05, *z*-ratio = .42, *p* = .67), although the language form had distinct influences on boys’ anticipated belonging. The masculine form significantly decreased boys’ anticipated belonging as they grew older (*b* = −0.14, *SE* = 0.06, *z*-ratio = −2.41, *p* = .016), although the pair form had no effect on their sense of anticipated belonging with increasing age (*b* = 0.12, *SE* = 0.07, *z*-ratio = 1.88, *p* = .06) (Fig 2a).

We also observed a significant Participant Gender x Occupation Stereotype interaction. Given that anticipated belonging concerns adolescents’ own anticipated sense of fit rather than their perception of others, we decomposed this interaction by comparing the different stereotype levels within each gender group. The interaction revealed differential effects of occupation stereotype between girls and boys, *F*(2, 261.96) = 50.68, *p* < .001, *R*²_β = .022, 90% CI [.018,.026]. (Fig 2b). While girls felt higher levels of anticipated belonging for feminine-stereotyped and gender-neutral occupations (Feminine: *M* = 3.42, *SD* = 1.11; Gender-neutral: *M* = 3.23, *SD* = 1.13; *p* = .455) than masculine-stereotyped occupations (*M* = 2.67, *SD* = 1.18; *p* < .001), boys anticipated similar levels of belonging for all occupations independent of stereotype (Feminine: *M* = 3.11, *SD* = 1.24; Masculine: *M* = 3.24, *SD* = 1.24; Gender-Neutral: *M* = 3.23, *SD* = 1.21; *ps* > .732). Moreover, the effect of occupation stereotype appeared to strengthen as girls grew older, as highlighted by the significant Participant Gender x Participant Age x Occupation Stereotype interaction, *F*(2, 261.89) = 3.14, *p* = .045, *R*²_β = .001, 90% CI [.00,.002] (Fig 2c). The interaction indicated that while girls’ sense of belonging did not differ between feminine and masculine stereotypical occupations as they grew older (*b* = 0.02, *SE* = 0.04, *z* = 0.45, *p* = .892), their sense of belonging increased for gender-neutral occupations with age, diverging significantly from both feminine (*b* = −0.085, *SE* = 0.032, *z* = −2.64, *p* = .023) and masculine-stereotyped (*b* = −0.105, *SE* = 0.039, *z* = −2.68, *p* = .020) occupations. In contrast, boys’ sense of belonging did not evolve with their age across different occupational stereotypes (all *ps* > .71).

### Discussion

In Experiment 1, the effect of language form, our main variable of interest, did not emerge as an independent effect, but rather through its interactions with participant characteristics (age and gender). In contrast, adolescents’ social perceptions and anticipated sense of belonging for different occupational groups were consistently shaped by occupational stereotypes. Taken together, these findings suggest that the role of language form in shaping adolescents’ gender representations may differ from patterns typically observed among adults.

For social perceptions, adolescents were susceptible to occupational stereotypes (supporting H1b). Individuals in feminine-stereotyped occupations were perceived as the warmest overall, although, contrary to expectations, not as less competent than those in masculine-stereotyped or gender-neutral occupations. Each gender group also rated individuals in occupations dominated by their own gender group as warmer than those in occupations dominated by the other gender, demonstrating in-group favouritism. For girls, this pattern extended to competence, with feminine-stereotyped occupations rated more competent than boys did. No such gender differences emerged for masculine-stereotyped occupations, most likely because these professions are already strongly associated with competence [46]. Critically, and contrary to H1a, neither warmth nor competence perceptions were modulated by the language form.

Adolescents’ sense of belonging was also affected by occupational stereotypes. Girls anticipated greater belonging in feminine-stereotyped and gender-neutral occupations (supporting H2b), whereas boys anticipated similar levels of belonging in all occupations, regardless of stereotype (contrary to H2b). Among girls, belonging in gender-neutral occupations increased with age, differing from other stereotypical occupations and potentially reflecting increased awareness that these jobs are open to both genders (supporting H3). Overall, this pattern suggests that girls may be more sensitive to stereotypes, whereas boys appear less influenced by them.

For our central variable, the one place language form appeared to have an influence was boys’ anticipated sense of belonging, with the masculine form decreasing their anticipated sense of belonging as they grew older, while the pair form showed an opposing, but non-significant trend (contrary to H2a). In contrast, girls’ anticipated sense of belonging was unaffected by the language form (contrary to H2a and H2c). While we cannot definitively determine from Experiment 1 alone the reasons why only boys demonstrated greater sensitivity to these language forms, a comparable asymmetry has been reported in adults with the pair form affecting men, but not women [48]. This effect of the pair form may be linked to boys’ greater sensitivity to linguistic forms, as found among men [29], or greater concerns about vocational choices. We return to this question of the effect of the pair form in Experiment 2, treating the present pattern as tentative as the pair form effect did not reach significance.

Taken together, the results of Experiment 1 raise two important questions. First, the effect of language form was only weakly evident and appeared only in interaction with participant gender and age, raising the question of the conditions under which the effect could emerge. Second, occupational gender stereotypes influenced both gender groups across all measures, albeit in different ways and to various extents, suggesting that individual characteristics may strongly affect how these sources of gender information may emerge. To better address these two issues, Experiment 2 considered two developmental factors relevant to adolescence: openness to GFL and career exploration stage. Evidence regarding openness to GFL remains mixed, with some studies suggesting that more positive attitudes are associated with a reduced visibility of women when processing the masculine form, whereas others indicate that this is true regardless of whether the masculine form or GFL forms are employed. Meanwhile, career exploration stages are particularly relevant during adolescence, as reflecting on one’s future vocational path can have direct consequences on vocational perceptions and influence how adolescents internalise information about different occupations. Building on this rationale, Experiment 2 sought to test the replicability of the findings and to extend them by assessing these individual differences.

## Experiment 2

Experiment 2 was a preregistered follow-up experiment designed to ensure the replicability of effects found in Experiment 1. We also extended our investigation to assess two exploratory variables related to individual differences: adolescents’ *career exploration* stages and *attitudes toward gender-fair language*. As we hypothesised that adolescents’ development would moderate the influence of occupational stereotypes and language form, we considered school year a more suitable predictor than chronological age for capturing the institutional context shaping adolescents’ vocational perceptions. We therefore directly compared the youngest (grade 9) and oldest (grade 11) groups within lower secondary school, using school year as a proxy for their developmental stage rather than age. Although these two school year groups represent a narrower age range than in Experiment 1, they allowed for comparisons to be situated within the same lower secondary school context.

### Method

#### Participants.

We recruited 196 participants from three lower secondary schools in Bienne, Switzerland, comprising 9^th-^ and 11th-grade students in the Swiss school system. None of these schools had participated in Experiment 1. Consent was obtained through the same procedure as Experiment 1, with two pupils opting out. We set the sample size to 65 pupils per grade to compare the two school year groups, unless the schools provided more pupils. As indicated in the preregistration, this sample size was intended to match the number of participants from each school year in Experiment 1. Participant recruitment and data collection were conducted between June 29 and August 23, 2022.

Following the same criteria as Experiment 1, we excluded five participants who marked ‘other’ for self-identified gender, one participant who was 18 years of age, and four participants with a *d’* (i.e., d-prime) score of 0 or below on the occupation recognition task (see *Procedures* section), indicating below chance-level performance. The final sample comprised 186 participants (86 girls, 100 boys; *M*_age_: 13.8; *SD*_age_: 1.36 years; age range: 12–17 years), with no significant age difference between girls and boys. There were 94 participants in grade 9 and 92 in grade 11.

#### Stimuli.

The stimuli consisted of the same occupations and descriptions used in Experiment 1.

#### Measures.

Perceived warmth, perceived competence, and anticipated sense of belonging. All response measures were identical to those used in Experiment 1, except for the perceived warmth and competence measures, which were rephrased to test the language form manipulation and to eliminate potential confounds more rigorously. In Experiment 1, the scales used the epicene term *personnes* [people] as a neutral way to refer to people without mentioning their gender, although *personnes* is grammatically feminine (2a). To better control for the potential grammatical effects of *personnes*, the occupations and adjectives were inflected by the grammatical form (i.e., masculine or pair forms) assigned to each participant (see 2b for the masculine form and 2c for the pair form condition).

(2a) *Ce sont des personnes*_*Gr.*_ _feminine plural_ *amicales*_*Gr.*_ _feminine plural_.
  [They are friendly _*Gr.* feminine plural_ people _*Gr.*_ _feminine plural_.]
(2b) *Les assistants sociaux*_*Gr.*_ _masculine generic_ *sont amicaux*_*Gr.*_ _masculine generic_.
  [The social workers _*Gr.*_ _masculine generic_ are friendly _*Gr.*_ _masculine generic_.]
(2c) *Les assistants sociales*_*Gr.*_ _feminine plural_ *et assistants sociaux*_*Gr.*_ _masculine plural_ *sont amicales*_*Gr.*_ _feminine plural_ *et amicaux*_*Gr.*_ _masculine plural_.
  [The social workers _*Gr.*_ _feminine plural_ and social workers _*Gr.*_ _masculine plural_ are friendly _*Gr.*_ _feminine plural_ and friendly _*Gr.*_ _masculine plural_.]

Cronbach’s alphas for the three measures were as follows: Warmth: feminine-stereotyped:.79, masculine-stereotyped = .82; gender-neutral = .83; Competence: feminine-stereotyped:.84, masculine-stereotyped = .86; gender-neutral = .87; Anticipated belonging: feminine-stereotyped:.84, masculine-stereotyped = .84; gender-neutral = .82.

Adolescent Attitudes Toward Gender-Fair Language Scale. We constructed the Adolescent Attitudes Toward Gender-Fair Language Scale (*GFL Scale*), inspired by the adult version of Tibblin et al. [15], to assess adolescents’ attitudes and knowledge regarding the pair form. The scale consisted of a brief explanation of the pair form, followed by two sets of items assessing two dimensions: participants’ knowledge and attitudes toward the use of pair forms. The Cronbach’s alphas were.80 for the knowledge and.55 for the attitude dimension. Due to the low internal consistency of the latter dimension, we removed one item, improving the score to.65, however, the reliability remained questionable. We thus refrain from reporting the secondary analysis involving the GFL scale hereon. Additional analysis including the GFL scale can be found in Supplementary Information 1 on the OSF project page.

Career Exploration Scale. To assess adolescents’ career exploration stages, we constructed a single-item question (i.e., *Where are you in your reflection and exploration of the job that you would like to do later?*). There were five statements, each representing an increasing degree of exploration (i.e., spanning from *1: I haven’t started thinking about possible jobs at all, and I have no idea what I want to do later on,* to *5: I have thought a lot about possible jobs that interest me and I have already decided what I want to do later on*.) Participants were instructed to select the statement that best reflected their current career exploration stage. Lower scores indicated early stages of career exploration, while higher scores indicated advanced stages.

#### Design.

The study took a mixed design with school year group (grade 9 vs. grade 11), participant gender (girls vs. boys), and language form (masculine vs. pair form) as between-participant variables, and occupational stereotype (feminine vs. masculine vs. gender-neutral) as a within-participant variable. Similar to Experiment 1, the dependent variables were perceived warmth, perceived competence, and anticipated sense of belonging.

#### Procedure.

The procedure was similar to that of Experiment 1, beginning with questions about participants’ background (e.g., gender, age), followed by the main questionnaire. As we planned to compare the different school year groups, the language form manipulation was equally distributed across gender groups in each school year (Grade 9: *n*_Pair–girls_ = 21, *n*_Masculine–girls_ = 19, *n*_Pair–boys_ = 27, *n*_Masculine–boys_ = 27; Grade 11: *n*_Pair–girls_ = 23, *n*_Masculine–girls_ = 23, *n*_Pair–boys_ = 23, *n*_Masculine–boys_ = 23).

After responding to the main questionnaire, participants completed an occupation recognition task to ensure they had read the list of occupations and descriptions properly. They selected six occupations they had seen in the questionnaire from 12 occupations. Among the 12 occupations, six were taken from the experimental stimuli and the remaining six were taken from Misersky et al.’s normed list [71]. The gender stereotypes of the experimental and filler items were equally distributed (i.e., two feminine-stereotyped, two masculine-stereotyped and two gender-neutral occupations for each item type). Participants then responded to the *GFL Scale* and the *Career Exploration Scale*.

#### Data analyses.

The main analyses deviated from the preregistered model-selection procedure, and instead took the same analytical approach as Experiment 1, essentially for the same reasons. We again also ran the preregistered analyses, which led to the same substantive conclusions as the analyses reported below (see OSF project page). One difference from Experiment 1 is that participant age was re-evaluated in terms of school year to better reflect developmental stages; consequently, school year was treated as a categorical predictor with sum-to-zero contrast coding (Grade 9 = 1, Grade 11 = −1).

### Results

#### Main analyses.

Perceived warmth. The critical language form variable was qualified by a four-way interaction with school year, participant gender, and occupation stereotype, *F*(2, 178) = 5.04, *p* = .007, *R*²_β = .001, 90% CI [.00,.003]. As this interaction was not predicted (nor preregistered), and because higher-order interactions are statistically underpowered, we report the interaction and its decomposition for completeness but interpret it with caution (i.e., could be a spurious interaction). Decomposing the interaction by school year, 9^th^ grade girls’ rated feminine-stereotyped and gender-neutral occupations similarly across language forms (Feminine_Pair_: *M* = 4.2, *SD* = .9; Feminine_Masculine_: *M* = 4.19, *SD* = .87, *p* = .926; Neutral-_Pair_: *M* = 3.76, *SD* = .94; Neutral_Masculine_: *M* = 3.69, *SD* = .91, *p* = .721). Consistent with the direction predicted by our hypothesis that the pair form would moderate the impact of occupation stereotypes (H1a), they tended to rate masculine-stereotyped occupations as warmer in the pair than the masculine form (Pair: *M* = 3.54, *SD* = .87; Masculine: *M* = 3.19, *SD* = 1.02), though this difference did not reach significance (*p* = .055). These differences between language forms did not emerge for Grade 11 girls or boys (all *ps* > .379).

**Fig 3:**
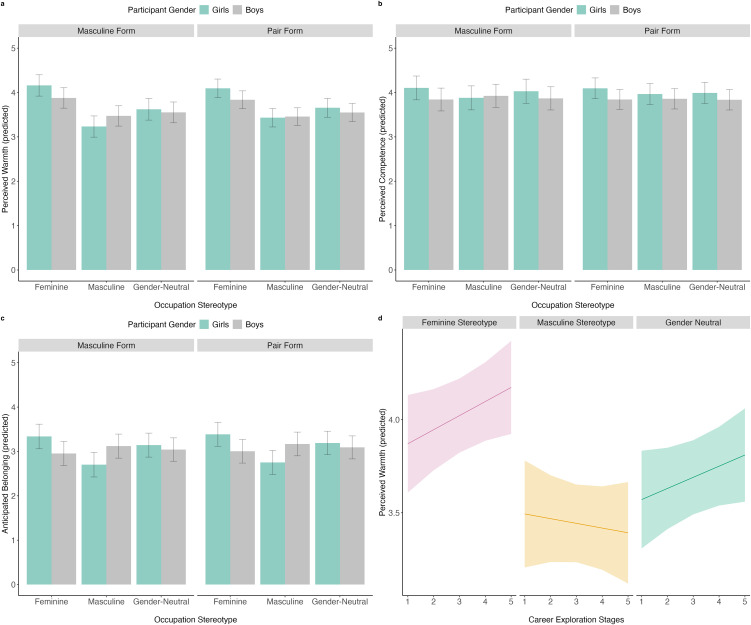
Predicted ratings from Experiment 2: (a) perceived warmth, (b) competence, and (c) anticipated belonging as a function of participant gender, language form, and occupation stereotype, and (d) perceived warmth as a function of grade 9 adolescents’ career exploration stages and occupation stereotype. Error bars represent confidence intervals.

Additionally, and as observed in Experiment 1, the main effect of occupation stereotype was significant, *F*(2, 13.9) = 15.32, *p* < .001, *R*²_β = .007, 90% CI [.055,.069] with individuals in feminine-stereotyped occupations being perceived higher in warmth than those in masculine-stereotyped (Feminine: *M* = 3.99, *SD* = .97; Masculine: *M* = 3.41, *SD* = .94; *p* < .001) and gender-neutral (*M* = 3.60, *SD* = .97; *p* < .001) occupations. Occupation stereotype also interacted with participant gender (*F*(2, 178) = 13.95, *p* < .0001, *R*²_β = .001, 90% CI [.005,.01]). As in Experiment1, girls perceived individuals in feminine-stereotyped occupations warmer than boys did (Girls: *M* = 4.12, *SD* = .87; Boys: *M* = 3.87, *SD* = 1.03; *p* = .001), and the two groups did not differ for gender-neutral occupations (Girls: *M* = 3.63, *SD* = .9; Boys: *M* = 3.58, *SD* = 1.03; *p* = .31). The pattern diverged only for the masculine stereotyped occupations; although boys rated them warmer than girls in Experiment 1, here, the two groups did not differ in their ratings (Girls: *M* = 3.33, *SD* = .87; Boys: *M* = 3.48, *SD* = .99; *p* = .11) (see Fig 3a).

Perceived competence. The main language-form variable was again included in a four-way interaction with school year, participant gender, and occupation stereotype. As this interaction was neither preregistered nor expected and given higher-order interactions of this kind are typically underpowered, we again interpret it with caution. This interaction, although retained in the model, did not reach significance, *F*(2, 178) = 3.00, *p* = .052, *R*²_β = .001, 90% CI [.00,.002]. Similarly, the lower-order School Year x Participant Gender x Occupation Stereotype interaction did not reach significance, *F*(2, 178) = 2.97, *p* = .054, *R*²_β = .001, 90% CI [.00,.002]. Decomposing the four way-interaction by school year revealed no differences between the language forms across participant gender and occupation stereotype (all *ps* > .5).

The Participant Gender x Occupation Stereotype interaction was significant, *F*(2, 178) = 6.69, *p* = .002, *R*²_β = .001, 90% CI [.001,.004] (Fig 3b). As in Experiment 1, girls rated individuals in feminine-stereotyped occupations as more competent than boys did (Girls: *M* = 4.09, *SD* = .89; Boys: *M* = 3.85, *SD* = 1.03; *p* = .005), while the two groups did not differ in ratings for individuals in masculine-stereotyped occupations (Girls: *M* = 3.92, *SD* = .95; Boys: *M* = 3.89, *SD* = 1.03; *p* = .735). The pattern of results between the experiments diverged only for gender-neutral occupations. Whereas girls rated them as more competent than boys in Experiment 1, here, the two groups did not differ (Girls: *M* = 4.01, *SD* = .91; Boys: *M* = 3.86, *SD* = 1.04; *p* = .1). No other main or interaction effects were significant (all *ps* > .09).

Anticipated sense of belonging. Language form produced no significant main or interaction effects. The analyses, however, did reveal a significant Participant Gender x Occupation Stereotype interaction *F*(2, 184.73) = 32.44, *p* < .001, *R*²_β = .018, 90% CI [.014,.022] (Fig 3c). As in Experiment 1, boys felt similar levels of anticipated sense of belonging for all occupation types (all *ps* > .054), whereas girls anticipated a greater sense of belonging to feminine-stereotyped (Feminine: *M* = 3.39, *SD* = 1.19; Masculine: *M* = 2.7, *SD* = 1.25; *p* < .001) and gender-neutral (Gender-neutral: *M* = 3.16, *SD* = 1.16; *p* = .008) occupations than masculine-stereotyped occupations (Masculine: *M* = 2.7, *SD* = 1.25). No other main or interaction effects were significant (all *p*s > .38).

#### Secondary analyses.

To further clarify whether the influence of occupation stereotypes and language forms can be attributed to increased knowledge of stereotypes resulting from career exploration and general GFL attitudes, both of which develop with time, we preregistered secondary analyses. Although we initially planned these analyses solely on sense of belonging, changes in the model selection procedure, prioritising the maximal model, revealed interactions in the warmth and competence measures that were not initially predicted. We therefore also decided to conduct secondary analyses on these measures if predicted interactions emerged in Experiment 1. These analyses aimed to test models that incorporated scores from the GFL Scale and the Career Exploration Scale. However, as noted earlier, the GFL Scale showed low reliability, and therefore, we refrain from reporting the results here. Further information on these analyses, as well as the descriptive statistics for both scales, can be found in the Supplementary Information on OSF.

Perceived warmth. Following the significant Participant Gender x Occupation Stereotype interaction emerging in the main analyses, we entered the Career Exploration x Occupation Stereotype interaction for each school year group. These analyses investigated whether adolescents’ level of career exploration would influence perceptions of warmth for individuals taking on specific gender-stereotyped occupations. A deeper exploration of career choices may deepen the understanding of occupation stereotypes, which are central to informing adolescents of their own suitability.

The secondary analysis indicated a significant Career Exploration x Occupation Stereotype interaction for 9^th^ graders, *F*(2, 91) = 3.4, *p* = .038, *R*²_β = .003, 90% CI [.002,.007]. While participants at the initial stages of career exploration viewed individuals equally warm across all occupation types (*ps* > .056), participants at the middle stages of career exploration perceived individuals in stereotypically feminine occupations higher in warmth than those in gender-neutral (*b* = .33, *SE* = .13, z-ratio = 2.60, *p* = .025) and masculine-stereotyped (*b* = .58, *SE* = .14, z-ratio = 4.30, *p* < .001) occupations. Participants at the most advanced levels of career exploration stages viewed individuals in feminine-stereotyped and gender-neutral occupations with the highest levels of warmth, although they still differed significantly from one other (*b* = .36, *SE* = .14, z-ratio = 2.60, *p* = .025) and from masculine-stereotyped occupations (Feminine: *b* = .78, *SE* = .16, z-ratio = 4.93, *p* < .001; Neutral: *b* = −.42, *SE* = .14, z-ratio = −2.91, *p* = .010) (see Fig 3d).

As for 11^th^ graders, a significant main effect of occupation stereotype (*F*(2, 16.73) = 15.29, *p* < .001, *R*²_β = .062, 90% CI [.053,.073]) revealed that individuals engaged in feminine-stereotyped occupations were seen as warmer than those in gender-neutral and masculine-stereotyped occupations (Feminine: *M* = 3.93, *SD* = .99; Masculine: *M* = 3.36, *SD* = .88; Gender-neutral: *M* = 3.48, *SD* = .97; *ps* < .0001), although individuals in gender-neutral and masculine-stereotyped occupations were perceived as equally warm (*p* = .48). Finally, a main effect of career exploration, (*F*(1, 90) = 123.41, *p* < .001, *R*²_β = .034, 90% CI [.027,.042]) indicated that perceptions of warmth globally increased as career exploration advanced. In sum, these results suggest that the effect of occupational stereotypes on younger adolescents’ warmth perceptions is linked to the extent to which they have explored potential career choices. Increased career exploration may deepen their understanding of occupational stereotypes and their suitability for these distinct jobs.

Perceived competence and anticipated sense of belonging. Following the significant Participant Gender x Occupation Stereotype interaction from the main analyses, we tested models with the main effects of career exploration, occupation stereotype, and their interaction separately for each participant gender group to examine whether career exploration could address the developing effects of occupation stereotype on perceived competence and sense of belonging.

No significant effects emerged for girls’ or boys’ perceptions of competence. In the boys’ competence model, career exploration was retained but did not reach significance. As for sense of belonging, a significant main effect of occupation stereotype for girls (*F*(2, 15.71) = 7.48, *p* = .005, *R*²_β = .053, 90% CI [.043,.064]) showed that their anticipated sense of belonging was higher for feminine-stereotyped and gender-neutral occupations than masculine-stereotyped occupations (*ps* < .024), whereas no significant effects emerged for boys. Across these secondary analyses, career exploration did not emerge as an explanatory factor, it was either non-significant when included or not retained during model selection. The preregistered analyses suggested the same conclusion regarding career exploration, although the main effect of occupation stereotype for girls reported here was not significant. In sum, these analyses indicated that career exploration did not account for the growing impact of occupation stereotypes on either perceived competence or anticipated sense of belonging.

### Discussion

The results of Experiment 2 largely corroborate the findings of Experiment 1. In contrast to the robust effects of occupational stereotypes, the effects involving language form were again small and did not reach significance.

Specifically, the effect of language form emerged in adolescents’ warmth and competence perceptions only within complex four-way interactions. With these effects being only marginally significant, not being preregistered, and likely underpowered, they should be interpreted cautiously and may reflect spurious findings (contrary to H1a). In contrast, occupation stereotype affected girls’ and boys’ perceptions of warmth (supporting H1b), with this effect further modulated by school year (supporting H3). Individuals in feminine-stereotyped occupations were generally perceived as warmer than those in other occupations, with girls rating them as warmer than boys. However, these gender differences did not extend to other occupations. Among early adolescents, these stereotype effects were partially accounted for by their career exploration stages (supporting H4), with those in middle and advanced stages of career exploration attributing higher warmth ratings for individuals in stereotypically feminine, and eventually gender-neutral occupations. As for competence perceptions, individuals in feminine-stereotyped occupations were not viewed as less competent than those in masculine or gender-neutral occupations (supporting Experiment 1 and contrary to H1b), although girls again rated them as more competent than boys did.

Finally, adolescents’ sense of belonging was not affected by language form (contrary to H2a). Girls anticipated greater belonging to feminine-stereotyped occupations than to masculine or gender-neutral occupations (supporting H2b) whereas boys felt an equal sense of belonging to all occupations, independent of stereotype (contrary to H2b). These findings may suggest that boys are generally less influenced by gender stereotypes, as was the case for Experiment 1.

## General discussion

In the present study, we assessed whether describing gender-stereotyped occupations in either the grammatical masculine or pair forms affects adolescents’ social perceptions (i.e., warmth, competence) and their anticipated sense of belonging to these occupations. As previous research has demonstrated that the pair form enhances mental accessibility to both genders in contrast to the grammatical masculine form, which emphasises men [14,15,30], we predicted that the pair form would globally increase perceived warmth and anticipated belonging while reducing perceived competence. Contrary to this prediction, language form played only a limited role. Across both experiments, occupational stereotype was the main source shaping adolescents’ social perceptions and anticipated sense of belonging, whereas the effect of language form was weak and inconsistent across different measures, emerging only in higher-order interactions that warrant cautious interpretation.

### The limited effects of gendered language forms in adolescents’ occupational representations

We expected that increasing the visibility of both women and men using the pair form would increase perceived warmth and decrease perceived competence for individuals in stereotypically masculine and gender-neutral occupations, and the opposite pattern for feminine-stereotyped occupations (H1a). Additionally, we also expected this manipulation to heighten girls’ anticipated sense of belonging (H2a). Our predictions, however, received little empirical support. When the effect of language form emerged, it was only evident within high-order interactions involving participant gender and age. Given that these interactions were underpowered and were not initially expected (i.e., preregistered), we have interpreted them as suggestive (contrary to H1a and H2a).

With this caveat in mind, the language-form effects that we found were not consistent across our two experiments. In Experiment 1, the masculine form decreased boys’ sense of belonging as they grew older, with the pair form showing no significant effects. In Experiment 2, the pair form increased warmth perceptions compared to the masculine form for masculine-stereotyped occupations among younger, grade 9 girls, although this effect was absent among boys and older, grade 11 girls. The language form effects that we observed thus varied in the measures (belonging, warmth), gender groups, and developmental stage which they were observed. These diverging and non-replicating effects reinforce our conclusion that language form may exert only a weak and unreliable influence on adolescents’ occupational representations (contrary to H1a, H2a).

Notably, these limited language form effects diverged from those reported by Vervecken et al. [31], who found consistent language form effects on perceived warmth and perceptions of women’s and men’s success. The differences may be due to methodological disparities. In their study, the experimenters read the occupations aloud to the participants, who received no additional details or descriptions. This meant that participants relied heavily on the ‘label’ of occupations to make their decisions, where different language forms could have exerted more significant effects on their representations. In contrast, participants in our study received more detailed descriptions of each occupation, potentially leading them to develop richer mental representations. Note that Escasain et al. [49] also found similar stereotype effects among adolescents but found no significant influence of the pair form on anticipated belonging using a paradigm that presented texts with occupational descriptions. In this respect, the manipulation of language form within a longer text may have been overridden or diluted by other gender-related content, and thus too minor to have exerted a significant effect. Overall, and contrary to adult research, the pair form did not consistently and uniformly render adolescents’ occupational representations more inclusive. Its influence was limited and secondary to the more robust effects of occupation stereotypes which we now turn.

### Influences of occupational gender stereotypes among girls and boys

In contrast to the limited role of language form, occupational stereotypes consistently affected adolescents gender representations (supporting H1b and H2b). Both girls and boys viewed individuals in feminine-stereotyped occupations as the warmest, compared with individuals in other occupations (supporting H1b). Notably, girls perceived individuals in these occupations as warmer than boys did, suggesting a degree of in-group favouritism for jobs stereotypically held by their gender (Experiments 1 and 2). This extended to their sense of belonging, with girls anticipating the greatest sense of belonging in feminine-stereotyped occupations (supporting H2b), while boys showed no specific preference (contrary to H2b). Such gender-group differences are consistent with prior work, including Tellhed et al. [56], who found that adolescents generally anticipate a stronger sense of belonging in careers dominated by their own gender and that girls reported greater self-efficacy in women-dominated careers, while boys demonstrated equal levels of confidence in both women- and men-dominated careers. These patterns suggested that girls are strongly affected by gender stereotypes from a young age, whereas boys were not affected by them to the same extent.

This difference mirrors several mechanisms reported in past studies. Girls and women face stereotype threat in men-dominated fields, where competence is generally associated with men, whereas men rarely encounter similar concerns [56]. Further adding to this issue, girls and women generally have lower expectations regarding self-efficacy than boys and men [82], which may narrow the range of careers they consider. The literature further confirms that beliefs about gendered traits contribute to gender segregation in employment, with feminine traits deemed essential for success in feminine-stereotyped jobs and masculine traits required in masculine-stereotyped jobs [46]. Our findings extend this literature to adolescence, demonstrating that even from a young age, girls vocational prospects and perceptions are influenced by gender stereotypes, while boys are encouraged to consider a range of occupations across various domains. Taken together, these findings reveal that occupational stereotypes impact vocational decision-making differently for girls and boys during this critical developmental period.

In addition, our study also suggested that individual factors can modulate adolescents’ susceptibility to these gender stereotypes. In line with H3, we found that knowledge of occupational stereotypes developed with adolescents’ age. As adolescents grew older, they increasingly viewed individuals in gender-neutral occupations as more competent and anticipated greater belonging to these occupations (Experiment 1). Similarly, older adolescents demonstrated an increased understanding of gender stereotypes compared to younger adolescents (Experiments 1 & 2), which led them to associate individuals in stereotypically feminine occupations more strongly with warmth. This development in stereotype knowledge among older adolescents was partially accounted for by their developing stages of career exploration (Experiment 2). These findings suggest that as adolescents consider potential career choices, their knowledge of occupations increases, which may modify or even intensify their stereotypical perceptions, at least for their perceptions of warmth (supporting H4). That career exploration did not account for the growing stereotype effects on perceived competence or anticipated belonging may suggest that regardless of whether individuals advance their knowledge of stereotypes through career exploration, adherence to stereotypes may be stable for these two measures.

### Implications, limitations, and future research

A notable limitation concerns our focal variable, language form. While we anticipated systematic effects of language form, these effects did not consistently emerge, nor did they provide support for our preregistered hypotheses. The influence of language form emerged only within complex interactions involving at least three variables, raising the possibility that these effects are spurious and did not replicate across experiments. In comparison, the effects of occupational stereotypes were robust and highlighted differences between the two gender groups.

These findings stand in clear contrast to past studies on adults, which consistently showed that the pair form leads to more gender balanced representations than the masculine form [14,15,29,30,48]. For adults, the pair form has been shown to activate more women exemplars in masculine-stereotyped domains [14,27] compared to the masculine form [30], although it does not exert a comparable effect for feminine-stereotyped domains [14,27]. These findings have typically been shown in experimental paradigms where occupational nouns were presented on their own [14,53], or within relatively short texts consisting of two or three sentences [14,28], whereas our study embedded them in longer texts. Interestingly, however, gender group differences among adults have not been demonstrated as consistently as in our adolescent sample, although, in general, women have been shown to activate and reference more women exemplars than men [30], a pattern also observed in our study.

One plausible account for our weak effects of language form may therefore be methodological, suggesting that these interventions do not exert strong enough effects when embedded within a longer text where other gender information is readily available. Considering that stereotype effects could overshadow the effect of language form, future research could test the effects of language forms with only gender-neutral occupations and in contexts where other gender-related information is minimal. A second, more probable explanation is developmental. Whereas among adults, gender information embedded in language plays a dominant role and can at times even override stereotype information [8], our adolescents showed that occupational stereotypes may act as the primary source of their mental representations. This difference highlights the distinctive developmental context of adolescence, a period in which individuals are driven by the need to construct their personal and social identities and may therefore be strongly influenced by salient social categories such as gender stereotypes [83]. While some research points to a growing level of flexibility in stereotype adherence during adolescence [41], our findings appear to show the contrary, particularly for girls who may more strongly conform to stereotypes than boys. This issue is particularly relevant and reflected in the gender group differences consistently observed across our different measures, indicating that occupational stereotypes influence girls and boys at this age in different ways [49]

A further consideration is the nature of our task. Unlike tasks that require explicit reflection on gender information, our study asked adolescents to evaluate individuals in specific gendered occupations and consider whether they would feel a sense of belonging in these positions. The process of considering realistic vocational careers and viewpoints is a much more subjective and personal decision that requires reflection on one’s gender-role identity and its alignment with the occupation. The differences in findings regarding the impact of GFL forms between adults’ and adolescents’ should thus be considered with this task difference in mind. In addition, a methodological difference between our experiments also warrants attention. In our study, Experiment 2 directly associated the attributes to each occupation, (i.e., Social workers are competent), whereas Experiment 1 did so indirectly (i.e., They are competent people), where *people* in French (i.e., *personnes*) is grammatically feminine and requires grammatical inflection. This may have introduced gender saliency in Experiment 1 that was absent in Experiment 2, resulting in some discrepancies in findings between the two experiments.

Finally, age was relevant to some of our findings (H3). Specifically, we found that the effects of language form differed and evolved as they grew older. These results appear to be related to their general development (H3), as well as adolescents’ consideration of career choices (H4), their general understanding of gender stereotypes, and potential exposure to different linguistic cues. Given the importance of age-related factors on adolescents’ gender representation, future research should focus on the developmental mechanisms affecting the intricate relationship between stereotypical information and language form.

These findings carry significant implications for educational practice and career guidance. First, given the extent of the influence of occupational gender stereotypes, schools may benefit from implementing more comprehensive efforts to expose adolescents to a broader range of professions. Such efforts should not only encourage adolescents to consider a broader range of potential vocational choices but also actively educate them to challenge social gender norms. In this regard, providing opportunities to meet role models who have pursued careers beyond traditional gender boundaries would be inspirational. As for language forms, although it may shape mental representations, our findings suggest that it may be weak relative to gender stereotypes. Thus, educational emphasis on inclusive linguistic forms should be understood as complementary to challenge occupational stereotypes, and preparatory for the eventual effects it may bring in adulthood.

## Conclusion

In sum, our study showed that adolescents’ vocational representations are influenced by multiple sources of gender information, including the gender of the individual, one’s knowledge of occupational gender stereotypes, and to a lesser extent, the language form in which occupations are described. The complex interactions between these sources of information differ as a function of adolescents’ age and career exploration stages. Considering that gender-related beliefs are heightened during adolescence, our findings provide insight into the different sources of information and their complex interactions that may distinctly impact girls’ and boys’ career choices.
